# Immunoprophylactic Potential of a New Recombinant *Leishmania infantum* Antigen for Canine Visceral Leishmaniasis: An *In Vitro* Finding

**DOI:** 10.3389/fimmu.2020.605044

**Published:** 2021-01-08

**Authors:** Rômulo Pessoa-e-Silva, Lays Adrianne Mendonça Trajano-Silva, Victor Vaitkevicius-Antão, Wagner José Tenório dos Santos, Franklin Barbalho Magalhães, Danielle Maria Nascimento Moura, Eiji Kevin Nakasone Nakasone, Virgínia Maria Barros de Lorena, Milena de Paiva-Cavalcanti

**Affiliations:** ^1^ Department of Microbiology, Aggeu Magalhães Institute, Recife, Brazil; ^2^ Tabosa de Almeida University Center, Caruaru, Brazil; ^3^ Department of Veterinary Medicine, Federal Rural University of Pernambuco, Recife, Brazil

**Keywords:** recombinant antigens, immunoprophylaxis, vaccinology, dog, visceral leishmaniasis

## Abstract

The development and application of safe and effective immunoprophylactic/immunotherapeutic agents against canine visceral leishmaniasis (CanL) have been pointed out as the only means for the real control of the disease. Thus, this study aimed to evaluate the *in vitro* cellular immune response of dogs, elicited by the new recombinant proteins of *Leishmania infantum*, Lci10 and Lci13, in order to investigate their potential for vaccinology. Twenty-four dogs were submitted to clinical, parasitological, serological and molecular tests, and then separated into two study groups: 12 infected (InD) and 12 non-infected dogs (NInD), and six of each group were directed for Lci10 and Lci13 evaluation. Peripheral blood mononuclear cells (PBMC) were cultured and stimulated with Lci10 (10 μg/ml) or Lci13 (5 μg/ml), and with *L. infantum* soluble antigen (LSA) (25 μg/ml) or no stimulus (NS) as controls. Afterwards, the mRNA levels of different cytokines were quantified through qPCR, and Nitric Oxide (NO) production was assessed in the culture supernatants. Significant differences were considered when p ≤ 0.05. The comparative analysis revealed that, in the NInD group, Lci13 promoted a significant increase in the expression of IFN-γ in relation to LSA (*p* = 0.0362), and the expression of this cytokine in NInD was significantly higher than that presented in the InD (*p* = 0.0028). A negative expression for TGF-β was obtained in both groups. Lci13 also induced a greater production of NO in relation to the NS sample in the NInD group. No significant differences were observed after stimulation with Lci10. In conclusion, the results suggest a protective role of Lci13 for uninfected animals, thus with a potential for immunoprophylaxis. The results will help to direct the antigen Lci13 for further studies (pre-clinical trials), in order to determine its immunogenicity and reactogenicity effects, as a way to consolidate its real applicability for vaccinology against CanL.

## Introduction

Canine Leishmaniasis (CanL) is a zoonotic disease caused by protozoan parasites of the genus Leishmania. It is considered endemic in more than 70 countries, and approximately 2.5 million dogs are infected just in south-western Europe (1, 2). As a result of a balance between host and parasite interaction, a great proportion of animals remain asymptomatic, but a significative number may develop severe signals and symptoms, such as hepatosplenomegaly and renal failure (3, 4). An effective diagnostic strategy, as well as prevention and treatment approaches for CanL are extremely important for controlling human visceral leishmaniasis (VL), since infected dogs are reservoirs of major epidemiological importance (5, 6). The association of mass immunoprophylaxis and a safe/feasible treatment for dogs from endemic areas has been recognized as one of the few strategies that can truly help in reducing VL cases (7–9). In Brazil, the control measure against CanL recommended by the Ministry of Health is the euthanasia of seropositive dogs, but so far this practice has not effectively reduced the number of human and canine cases (10–13).

Immunization and treatment of dogs may prevent the transmission of Leishmania to the insect vector, due to a decrease of parasite load in their skin (8, 14). Currently, only three vaccine formulations are available for dogs, being two of them produced in Europe: Canileish^®^ (Virbac, France) and Letifend^®^ (Laboratorios Leti, Spain) (15). In Brazil, the Leish-Tec^®^ is the sole immunoprophylactic vaccine available against CanL, and despite its efficacy of 71.4% (16), it is still expensive, considering the vaccination protocol of three doses plus boost, thus making it inaccessible for the poorest and hard to be adopted in national immunization campaigns.

As for therapy, some few drugs are available for treatment of infected dogs throughout the world, but the possibility of not providing a parasitological cure, and the significant side effects (17, 18) raise the need for alternative formulations, especially immunomodulatory drugs which might be associated with anti-Leishmania medicines. For therapeutic success, it is believed that there is a dependence, at least in part, on changes in the host’s immune response against the parasite (19, 20). In this context, the advances in the vaccinology field have motivated the search for new antigenic candidates, especially recombinant proteins (21–23).

To ensure a successful immunoprophylactic/immunotherapeutic formulation, the antigens studied must be able to immunomodulate the cell-mediated response, inducing a strong and long-lasting Th1 immunity, with a predominant production of proinflammatory cytokines, such as IFN-γ and TNF (24). These cytokines enable the production of leishmanicidal factors by infected macrophages, as Nitric Oxide (NO) and Reactive Oxygen Species (ROS), thus preventing the establishment of infection and controlling the progression of the disease (25). In contrast, a predominant Th2 response, associated especially with the increasing of IL-4 and TGF-β levels in the tissue microenvironments, carries to inhibition of NO synthesis by macrophages, thus favoring parasite survival (26, 27).

A group of new *Leishmania infantum* recombinant antigens have been previously described as having a great potential for their use in the detection of anti-Leishmania antibodies for the diagnosis of human and canine VL forms. Some of these antigens have promoted good sensitivity and specificity rates (ranging from 77 to 100%) when sera from infected and healthy dogs were tested using Enzyme-linked Immunosorbent Assay (ELISA) (23, 28). Therefore, these results support the execution of studies for the evaluation of these recombinant proteins as vaccine candidates. Two of these were the Lci10 (accession number: LinJ.34.2360) and Lci13 (accession number: LinJ.30.2820). The Lci10 orthologue in T. brucei was characterized as a flagellar attachment zone protein (29). In *Leishmania*, this protein sequence was shown to be composed of repetitive domains varying from 68 to 198 amino acids (23). The Lci13 was identified as a homolog of the 70-kDa mitochondrial heat shock protein (mHSP70) in L. infantum (30).

In this study we investigated the immunoprophylactic and immunotherapeutic potential of the recombinant Lci10 and Lci13 antigens against CanL by assaying their ability to induce gene expression of selected cytokines and NO synthesis in canine PBMC cultures from infected and non-infected dogs. Our results provide scientific subsidies that may direct studies for the development of new vaccines.

## Materials and Methods

### Sampling

A convenience sampling was adopted (31). Twenty-seven dogs of different breeds and ages between 1 and 7 years, both sexes, from domicile or peridomicile were included in the study. Three infected animals were arbitrarily chosen to be employed for the definition of optimal culture conditions and 24 were distributed as “Infected Dogs” (InD - 12) and “Non-Infected Dogs” (NInD - 12), for the evaluation by study groups.

Dogs with a compatible epidemiological history, with or without clinical characteristics of CanL and with two or more positive tests (serology, qPCR, parasitological) were included in the InD group. Animals were included in the NInD group when all tests were negative, and if they presented no clinical signs/symptoms suggestive for CanL.

Dogs included in the study were from the Zoonosis Control Unit (*Unidade de Controle de Zoonoses* - UCZ), located in the city of Caruaru (Lat. 08° 16’53” S, Lon. 35° 58’25” W), a mesoregion in Pernambuco state, and from Camaragibe city (Lat. 08° 1’14” S, Lon. 34° 58’54” W), Pernambuco state, Brazil.

### Clinical Evaluation and Sample Collection

Dogs were clinically evaluated, and clinical signals/symptoms suggestive for CanL were recorded: weight loss, hepatosplenomegaly, lymphadenomegaly, ulcerative/seborrheic dermatitis, generalized or localized alopecia, onychogryphosis, cardiorespiratory changes, mucosal bleeding/secretions and neurological changes. Whole blood was collected from each animal, by venipuncture, using different collection tubes: 18 ml in heparin tubes, to carry out the separation of Peripheral Blood Mononuclear Cells (PBMC) and cell culture, 4 ml in a dry tube and 4 ml in tubes with Ethylenediaminetetraacetic Acid (EDTA), for serological and molecular tests, respectively.

For animals whose collection procedure included bone marrow and/or lymph node aspiration, atropine was applied at a dose of 0.044 mg/kg as preanesthetic medication (PAM). After PAM, dissociative anesthesia was carried out, and after 5 min of latency (32), bone marrow puncture at the crest of the sternum bone (20 ml syringe, 40×1,20 mm needle) and/or popliteal lymph node puncture (10 ml syringe, 25×0,7 mm needle) were started.

### Laboratorial Diagnosis

Parasitological test was based on the search for amastigote forms in stained smears, prepared from medullary or lymph node aspirate, using an optical microscope (1,000× magnification). The serological test was performed using the immunochromatographic rapid test Dual Path Platform (TR-DPP^®^ Bio-Maguinhos/FIOCRUZ, Rio de Janeiro, RJ, BR). Serum samples were also tested with an ELISA based test (EIE-LVC Bio-Manguinhos/FIOCRUZ, Rio de Janeiro, RJ, BR), both tests adopted by the Brazilian Ministry of Health to determine the positivity of dogs in the active searches of CanL and performed according to the manufacturer’s instructions. The serology was considered “positive” only if both TR-DPP^®^ and EIE-LVC were reagent, and it was considered “negative” only if both tests were no reagent. The molecular test was based on the *L. infantum* kinetoplast DNA (kDNA) minicircle detection, using real time quantitative Polymerase Chain Reaction (qPCR), as previously described by Paiva-Cavalcanti et al. (33), and DNA was extracted from EDTA whole blood using QIAamp^®^ DNA Blood Mini Kit (QIAGEN^®^ Sample and Assay Technologies, Germantown, MD, USA), according to the manufacturer’s instructions. Parasitological and molecular analyses were carried out at Aggeu Magalhães Institute (IAM)/FIOCRUZ. Serological tests were executed and provided by the *Laboratório da IV Gerência Regional de Saúd*e – Caruaru, Pernambuco, Brazil.

### Production of Recombinant Antigens

Expression and purification of the His-tagged recombinant antigens Lci10 and Lci13 was performed as described (23), using *Escherichia coli* BL21(DE3) pLysS cells (Invitrogen^®^, Carlsbad, CA, USA) transformed with pRSET-derived plasmids containing the Lci10 or Lci13 genes. Nickel-nitrilotriacetic acid (Ni-NTA) Agarose (QIAGEN^®^, Germantown, MD, USA) was used for purification. Protein concentrations were determined by the Bradford’s method (34). SDS-PAGE gels (15%) were performed to check the effectiveness of each purification.

### Production of *L. (L.) infantum* Antigenic Fractions

*L. infantum* soluble antigenic fraction (LSA) was obtained from promastigote forms of *L. infantum* (strain MHOM/TN/1980/IPT1), following the protocol standardized by Gonçalves-de-Albuquerque et al. (35). The protein concentration was determined using the Bradford’s method (34). Thereafter, the LSA was aliquoted and stored at −80°C until its use in cell culture assays.

### PBMC Isolation and Cell Culture

PBMCs were obtained from heparinized blood using a protocol based on density centrifugation and employing Ficoll-Paque™ (GE Healthcare, Uppsala, SWE), as described by Lorena et al. (36). The cell pellet containing the mononuclear cells was resuspended in RPMI 1640 medium and counted in the Neubauer chamber using Trypan blue to check viability. The cell suspension was adjusted to the concentration of 1x10^6^ cells per ml of RPMI 1640 medium supplemented with 10% of fetal bovine serum (FBS). The cells were placed in 24-well plates of polystyrene and then incubated at 37°C in the presence of 5% CO_2_; the culture time was established as described in *Standardization of Culture Time and Antigens Concentrations*.

### Cytokines Gene Expression Analysis

#### Standardization of Culture Time and Antigens Concentrations

After blood collection, a pool of PBMCs from three positive animals was made and then it was plated (1×10^6^ cells per well). The cells were subsequently stimulated with different concentrations of the recombinant antigens (2.5, 5 and 10 μg/ml) and with LSA (25 μg/ml). A negative control was also included. For each stimulus, two wells were used as a way of ensuring that enough sample for qPCR would be obtained. The plates were incubated for 24, 48, and 72 h at 37°C and 5% CO_2_. The pool was used to ensure that all stimuli were evaluated with a same cell population in three different culture times. The choice of the best culture time and antigen concentration was assessed by evaluating gene expression of Interferon gamma (IFN-γ); Tumor Necrosis Factor (TNF), Interleukin 2 (IL-2), IL-4, IL-10, and Tumor Growth Factor beta (TGF-β), by Relative Quantitation (RQ) using qPCR *(Evaluation of Cytokines mRNA Expression by qPCR)*. This optimization step aimed to avoid the use of a culture condition that was not enough to induce a detectable cytokine gene expression. A positive response for the resistance profile (Th1), with suppression of the susceptibility profile (Th2) was also employed as criteria to choose the times and concentrations.

#### Evaluation of Cytokines mRNA Expression by qPCR

Total RNA was extracted from the PBMC cultures using TRIzol^®^ (Invitrogen^®^, Carlsbad, CA, USA) and transformed into cDNA through Reverse Transcription qPCR (RT-qPCR), using the TaqMan™ Reverse Transcription kit. After dosing in a spectrophotometer, cDNA levels for the cytokines IFN-γ (ID: Cf02623316_m1 – 117bp); TNF (ID: Cf02691119_s1 – 137 bp); IL-2 (ID: Cf02623318_m1 – 107 bp); IL-4 (ID: Cf02623271_s1 – 110 bp); IL-10 (ID: Cf02624264_m1 – 114 bp) and TGF-β (ID: Cf02623326_m1 – 124 bp) were quantified by qPCR (TaqMan^®^ System) with the ABI-PRISM 7500 sequence detection system and using the TaqMan^®^ Gene Expression PCR kit Master Mix (Applied Biosystems™, Foster City, CA, USA). The endogenous control applied for the reaction was the Dog Glyceraldehyde 3-Phosphate Dehydrogenase (GAPDH) gene (ID: Cf04419463_gh – 54 bp) (Applied Biosystems™). The RQ was calculated after normalization of the samples with the endogenous control by the comparative threshold cycle (Ct) method, and negative control (no stimulus) was employed as reference sample. All samples were analyzed in triplicates.

### Evaluation of Nitric Oxide Production

To have a better understanding of the immune response elicited by Lci10 and Lci13, NO production was determined indirectly by nitrite dosage in the supernatants of the PBMC cultures from both groups, after stimulation with the recombinant proteins, using Griess reagent, according to Resende et al. (37). The supernatants were collected before RNA extraction process and submitted to freezing (−80°C) until dosage.

### Evaluation of the Recombinant Antigens by Study Groups

After defining the best culture time and concentrations of the new antigens, PBMCs from the dogs of both study groups were stimulated as follows: Lci10, Lci13, LSA (25 μg/ml) (38, 39), and negative control (no stimulus). The mitogen phytohemagglutinin (PHA – 5 μg/ml) was used as a control for the culture of PBMC. It was followed by the evaluation of gene expression of IFN-γ, TNF, IL-10, and TGF-β, as well as by the evaluation of the production of NO in the supernatants as described in *Evaluation of Cytokines mRNA Expression by qPCR* and *Evaluation of Nitric Oxide Production*, respectively. The data obtained with the cell cultures stimulated with the antigens were considered only after the observation of positive gene expression for some of the evaluated cytokines induced by PHA, then confirming the viability of the cells and adequacy of the general culture conditions. Twelve dogs from each group were randomly divided to evaluate the recombinant antigens. Thus, two subgroups of six animals were formed, and each subgroup was used to evaluate one recombinant antigen through gene expression analysis. All data obtained with the groups were expressed as the mean ± standard deviation (t-test) or the median with interquartile range (Mann-Whitney test).

### Comparative Analyses

After the Kolmogorov-Smirnov normality test, the t-test (parametric) was used for the samples that obtained normal distribution, and for the samples with non-normal distribution, the Mann-Whitney test (non-parametric) was used. Correlation between the cytokines was performed using the Spearman test for non-normal samples and the Pearson test for normal samples. All tests were performed using GraphPad Prism 5 (Graphpad Software Inc. 2007, San Diego, CA, USA), and differences were considered statistically significant when p ≤ 0.05.

A table summarizing all the immune response analyzes obtained with the two recombinant antigens was constructed. The results were expressed in “plus” signs, as follows: (+): the protective potential or potential for vaccinology observed is only suggestive, with no statistically significant differences; (++): the protective profile or potential observed is indicative and there are one or more statistically significant differences in the analysis; (*): there was no suggestive or indicative protective profile in the analysis. The final number of “plus” signs (score) indicates the conclusive major potential (immunoprophylactic or immunotherapeutic) of each recombinant protein.

### Ethical Considerations

The owners were invited to participate in the research and to sign an informed consent form authorizing the use of the material collected for scientific purposes. The project was approved by the Ethics Committee on Animal Use of the Aggeu Magalhães Institute (n° 76/2014).

## Results

### Lci10 and Lci13 Description and Purification

Purified recombinant antigens were previously visualized through SDS-PAGE (23). The *in silico* prediction of Lci10 and Lci13 molecular weight is 35 and 45 kDa, respectively. However, the approximated molecular weight of Lci10 in gel is quite different (higher than 35 kDa), but constant in all purifications performed for this study, confirmed through SDS-PAGE (data not shown).

### Definition of Culture Time and Antigens Concentrations

**Figure 1:**
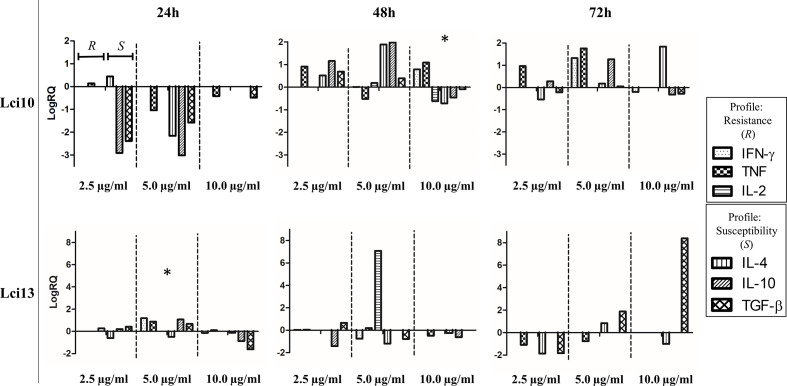
Overview of cytokine gene expression profiles obtained after stimulation of peripheral blood mononuclear cells (PBMCs) from positive animals with recombinant antigens in different concentrations and different culture times. The culture conditions were chosen based on the resistance and susceptibility profiles presented. The values are expressed in logarithmic scale. Values of cytokine gene expression obtained after the different stimulus are expressed positively or negatively in relation to control (sample without stimulus), which is adjusted to zero. RQ, relative quantitation. * conditions chosen for recombinant antigens.

PBMC stimulation with Lci10 in the concentration of 10 µg/ml during 48 h was the best among the conditions tested, presenting a predominant Th1 profile. For Lci13, the concentration of 5 μg/ml stimulated a balance between Th1 and Th2 profiles after 24 h of culture, which did not occur in the other conditions tested (**Figure 1**). The comparison between the immune profile obtained in the conditions chosen for both antigens and the LSA is presented in the **Supplementary Figure**.

### Evaluation of the Recombinant Antigens in the Study Groups

#### Comparative Evaluation Between the Recombinant Antigens and Soluble Leishmania Proteins

**Figure 2:**
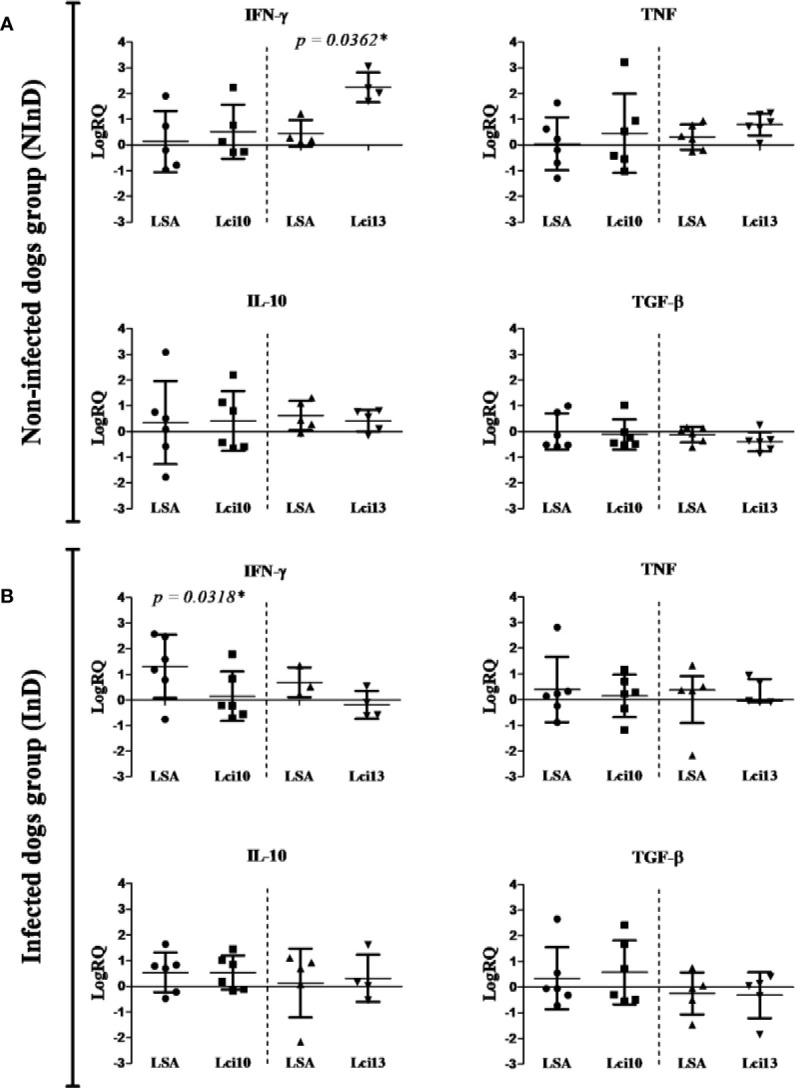
Cytokines gene expression in the non-infected dog (NInD - **A**) and infected dog (InD - **B**) groups, after stimulus of peripheral blood mononuclear cells (PBMCs) with LSA/Lci10 or LSA/Lci13. The values are expressed in logarithmic analysis (log in base 10), with mean ± standard deviation (t-test) or median with interquartile range (Mann-Whitney), obtained from the analysis of four to six dogs. The sample without stimulus is equal to zero (reaction calibrator), and the other values are expressed positively or negatively. LSA, *L. infantum* soluble antigen. RQ, relative quantitation. In some charts, the number of dogs per stimulus was less than six because the amplification of the reference sample failed in some few cases, thus not being possible to calculate the RQ. *significant difference.

Recombinant antigens were initially compared with *L. infantum* soluble antigen (LSA), through the analysis of the immune response elicited by the stimuli in each study group, NInD and InD. Parallel experiments were thus carried out with a set of samples evaluated with LSA/Lci10 and the second set with LSA/Lci13. The NInD group was the first analyzed (**Figure 2A**). Overall, both antigens and LSA stimulated expression of IFN-γ, TNF, and IL-10, when compared with non-stimulated cells (represented in the graphs of **Figure 2** as the “zero” line), and a slight decrease on the expression of TGF-β was observed. For the first antigen tested, Lci10, no significant difference in expression was observed for the four cytokines assayed, when cells stimulated with this protein were compared to those stimulated with LSA. In contrast, for Lci13, a significant higher expression of IFN-γ was observed for the cells stimulated with this antigen in comparison to the LSA stimulated cells (LogRQ LSA × Lci13: IFN-γ = 0.44 × 2.24, with *p* = 0.0362), although no differences were observed with the other three cytokines.

The results for the InD group are summarized in **Figure 2B**. No significant differences in stimulation between Lci10 and the LSA sample were observed for three of the cytokines, TNF, IL-10 and TGF-β. In contrast, a significant lower expression of IFN-γ induced by Lci10 was observed in comparison with LSA (LogRQ LSA × Lci10: IFN-γ = 1.30 × 0.15, with *p* = 0.0318). No statistically significant differences in expression of the four cytokines were observed between cells stimulated with Lci13 or LSA. Besides, a lower and negative expression of IFN-γ is clearly noticed (LogRQ LSA × Lci13: IFN-γ = 0.69 × −0.19, with *p* = 0.2286).

#### Immunoprophylactic Potential × Immunotherapeutic Potential of Lci13

**Figure 3:**
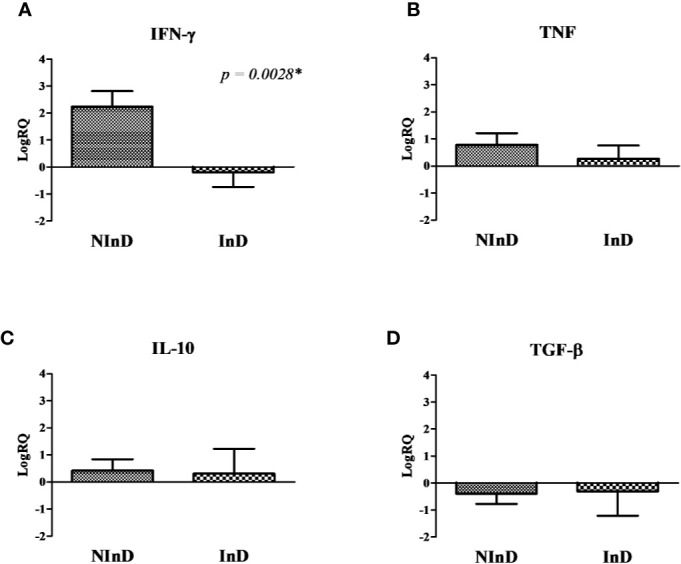
Cytokines gene expression in the two study groups after stimulus of peripheral blood mononuclear cells (PBMCs) with Lci13. The values are expressed in logarithmic analysis (log in base 10) and they were plotted based on the results shown in **Figure 2**. **(A)** Mean of RQ values of Interferon gamma-γ (IFN-γ), **(B)** Tumor Necrosis Factor (TNF), **(C)** IL-10, and **(D)** Tumor Growth Factor beta (TGF-β). NInD: non-infected dogs. InD, infected dogs. RQ, relative quantitation. *significant difference.

To better evaluate the Lci13 in relation to its potential for vaccinology, the data from the NInD and InD groups were compared (**Figure 3**).

Lci13 induced a marked different profile in the two groups, with higher expression of IFN-γ in the NInD, and with a suppression in the InD (LogRQ NInD × InD = 2.24 × −0.19, with *p* = 0.0028) (**Figure 3A**). In addition, a suppression in TGF-β expression was observed for both groups (**Figure 3D**). There were positive expressions of TNF and IL-10 in both groups, and no statistical differences were observed (**Figures 3B, C**).

According to the results obtained in the group with healthy animals (NInD), Lci13 has a predominant immunoprophylactic potential, causing a higher expression of pro-inflammatory cytokines, and reduced TGF-β mRNA levels in comparison to the sample without stimulus.

NInD and InD groups stimulated with Lci10 were also compared, but no significant differences were obtained (data not shown).

#### Correlation of Cytokines After Stimulation With Lci10 and Lci13

Analyzing the correlation between cytokines expressed after stimulation with Lci10, there was a significant positive correlation between IFN-γ and TNF (*p* = 0.011) in the NInD group, with a correlation coefficient considered strong (r = 0.96).

After stimulation with Lci13, a significant negative correlation occurred between IFN-γ and TGF-β (*p* = 0.03) in the InD group, with a correlation coefficient considered strong (r = −0.97).

#### Evaluation of Nitric Oxide Production

**Figure 4:**
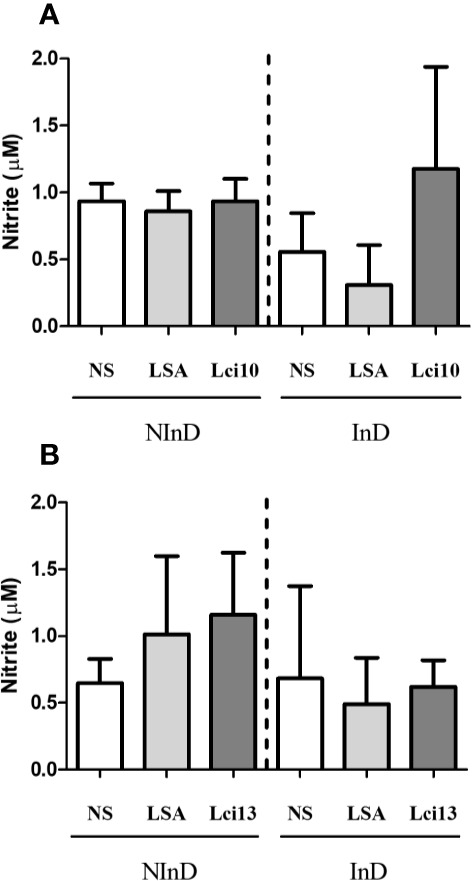
Evaluation of NO production induced by Lci10 **(A)** and Lci13 **(B)** in the cultures of peripheral blood mononuclear cells (PBMCs) from infected and non-infected dogs. The NO production was determined indirectly by nitrite dosage. The values are expressed as mean ± standard deviation obtained from the analysis of 24 dogs, being 12 dogs for each protein, and six per study group. LSA: *L. infantum* soluble antigen; NS, no stimulus.

In the NInD group, Lci10 promoted similar NO production in relation to LSA and ‘no stimulus’ (NS), while in the InD group, the mean value of NO production was higher for Lci10 antigen when compared to NS and LSA (**Figure 4A**). Lci13 was able to promote a higher NO production when compared to NS (*p* = 0.0605) and LSA (*p* = 0.3634) in the NInD group. In the InD group, NO production by Lci13 was quite similar to that obtained in NS and LSA, and lower than the presented by Lci13 in the NInD group (**Figure 4B**). For both antigens, there were no statistically significant differences.

### Global Evaluation of Lci10 and Lci13 Potential for Vaccinology Against CVL

**Table 1** shows the immune response analyzes performed with the two recombinant antigens, and the major potential in vaccinology obtained by each one. Thus, from the results achieved in the NInD group, the Lci13 showed an immunoprophylactic potential, with a score of five “plus” signs, presenting a more prominent potential in relation to Lci10, which obtained a score of two “plus” signs.

**Table 1:** Global assessment of immune response analyzes performed after canine peripheral blood mononuclear cell (PBMC) stimulation with *L. infantum* recombinant antigens and the conclusion about the potential in vaccinology for CVL.

Recombinant antigen	Gene expression analysis		Correlation between cytokines (strong, with p ≤ 0.05)	Nitric Oxide (NO)	Potential for vaccinology CONCLUSION
	Recombinant antigenXLSA	Immunoprophylaxis (NInD)XImmunotherapy (InD)			
**Lci10**	Th1 NInD group (+) (**Figure 2A**)	*	IFN-γ x TNF (POS - Th1) NInD group (+)	*	NInD: ++ (02) InD: 0 SUGGESTIVE IMMUNOPROPHYLAXIS, ONLY.
**Lci13**	Th1 (↑IFN-γ)NInD group (++) (**Figure 2A**)	(↑IFN-γ) NInD group (++) (**Figure 3A**)	IFN-γ x TGF-β (NEG - Th1) InD group (+)	Th1NInD group (+)(**Figure 4B**)	NInD: +++++ (05) InD: + (01) IMMUNOPROPHYLAXIS.

(+): the protective profile or potential for vaccinology observed for the recombinant antigen is only suggestive, with no statistically significant differences; (++): the protective profile or potential for vaccinology observed for the recombinant antigen is indicative and there are one or more statistically significant differences in the analysis; (*): there was no suggestive or indicative protective profile for the recombinant antigen in the analysis. For the correlation analysis between cytokines, one “plus” sign (+) was considered in the occurrence of a strong and significant positive/negative correlation. LSA, L. infantum soluble antigen; POS, positive correlation; NEG, negative correlation.

## Discussion

Immunogenic proteins with capability to stimulate a predominantly pro-inflammatory response are considered interesting candidates to be further investigated for immunoprophylaxis and immunotherapy for CanL (24, 40–42). In our study, we evaluated the potential of two new recombinant antigens from *L. infantum*, by stimulating cultures of PBMC belonging to healthy and infected dogs.

After comparing the means obtained in gene expression analyses, Lci13 results show that this protein presents a potential for immunoprophylaxis, since a predominant Th1 immune profile in the NInD group was observed. Lci13 induced a significant higher expression of the pro-inflammatory cytokine IFN-γ in relation to LSA in the NInD group, and this gene expression was also significantly higher in relation to the levels obtained in the InD group. Studies indicate that resistance to *L. infantum* infection in dogs is owed to a strong Th1 response, mainly due to IFN-γ, responsible for stimulating cell proliferation and, together with TNF, for activating macrophages and other cells of the immune system, leading to parasite destruction by increasing intracellular NO levels (4, 40, 43, 44). The expression for TNF, IL-10 and TGF-β was very similar between groups, but gene expression for the regulatory cytokine TGF-β in both groups presented a negative mean in relation to sample without stimulus.

Lci13 induced a positive expression for IL-10 in the NInD group. The role of IL-10 in the pathogenesis of CanL is still controversial. According to some authors, this cytokine, together with IL-4 and TGF-β, favors parasite proliferation, through inhibition of the production of IFN-γ and TNF, besides being responsible for the proliferation and differentiation of B cells, allowing greater activation of Th2 profile (24, 45). In contrast, other authors affirm that IL-10 has an important regulatory role in the immune response against CanL. For example, Maia; Campino (46) indicate that the balance between IL-10 and TNF is associated with reduction of parasite load in different tissues such as skin and spleen, and also with the absence of clinical signs in infected animals. Furthermore, Solano-Gallego et al. (47) performed IL-10 dosage from the supernatants of whole-blood cultures from dogs with different clinical statuses (from I – mild disease, to IV – very severe disease). The cultures were stimulated with LSA, and they concluded that IL-10 concentration was not associated with disease severity. Hence, the gene expression results presented here support that the Lci13 may be an interesting target to be further evaluated using murine models to determine its true immunoprophylactic potential.

The correlation analyses between the cytokines produced after stimulation with Lci13 displayed a significant negative correlation between IFN-γ and TGF-β in the InD group, demonstrating that an increase in IFN-γ levels will probably be accompanied with a decrease of TGF-β levels. Nonetheless, the mean value of gene expression obtained for IFN-γ in this group was negative, what highlights the importance to further evaluate the real applicability of Lci13 for immunotherapy, employing different tools, such as ELISA and flow cytometry.

In the NInD group, the NO dosage indicated that Lci13 helped mononuclear cells in the culture to increase the levels of this leishmanicidal factor. This finding is in accordance with the data obtained in the gene expression analyses, since the greater expression of both proinflammatory cytokines (IFN-γ and TNF) obtained in the NInD group may have stimulated a higher NO production through a synergistic induction.

In relation to Lci10, the mean gene expressions of IFN-γ and TNF stimulated by this recombinant protein were positive and higher when compared to those stimulated by LSA, while IL-10 and TGF-β expressions were quite similar to those presented by the soluble antigen. Comparing NInD and InD groups, it was evidenced that the antigen Lci10 was able to induce a positive and a higher gene expression of cytokines from the Th1 profile in the NInD group (data not shown). Nevertheless, no statistical differences were obtained, and a possible immunoprophylactic potential need to be further investigated through other *in vitro* assays before tests in animal model.

The correlation analyses between the cytokines produced after stimulation with Lci10 in the NInD group showed a significant positive correlation between IFN-γ and TNF, with a strong correlation coefficient, and this concurrent increasing of Th1 cytokines in the culture reinforces a possible protective potential of this antigen for healthy animals. However, it is important to notice that differences in NO synthesis were not detected in this group when compared to NS or LSA.

Finally, some points of the analyses must be commented. Different infections/coinfections may occur in animals from peridomicile and even from domicile, and are sometimes very difficult to detect, even with a rigorous clinical evaluation (48). However, those animals exposed to different infections (parasitic or non-parasitic) will be the targets of a potential mass vaccination program in the future, so it is also important to conduct initial studies with a population subjected to these conditions. In addition, we believe that *in vitro* assays performed by using immunogenic proteins are able to signal the immune profile obtained by the stimulated cells, thus helping to direct the best(s) target(s) to subsequent stages of vaccinology study. Nevertheless, a stronger immune response should be obtained when using the new proteins together with adjuvants during *in vivo* studies.

In conclusion, the Lci13 showed to be a promising antigen, with a protective response elicited in the group of healthy dogs. This recombinant antigen will be further evaluated in relation to its immunogenicity and reactogenicity in *in vivo* assays (pre-clinical trials), in order to consolidate its applicability as a vaccine candidate. The Lci10 showed a suggestive immunoprophylactic potential, but further assays need to be performed *in vitro* to confirm this finding, before the execution of possible *in vivo* assays.

## Data Availability Statement

The original contributions presented in the study are included in the article/**Supplementary Material**. Further inquiries can be directed to the corresponding author.

## Ethics Statement

The animal study was reviewed and approved by Ethics Committee on Animal Use of the Aggeu Magalhães Institute (n° 76/2014). Written informed consent was obtained from the owners for the participation of their animals in this study.

## Author Contributions

RP-e-S planned/executed the experiments, analyzed the data and wrote the manuscript. LT-S and VV-A planned/executed the experiments as well as analyzed the data. WS, FM, and DM were responsible for the production of the recombinant antigens and for the analysis of data. EN collected biological samples, performed clinical evaluation, and planned/executed the experiments. VL and MP-C planned some experiments, contributed substantially to the data analysis, and assisted in the writing of the manuscript. All authors contributed to the article and approved the submitted version.

## Conflict of Interest

The authors declare that the research was conducted in the absence of any commercial or financial relationships that could be construed as a potential conflict of interest.

## References

[1] Baneth G Koutinas AF Solano-Gallego L Bourdeau P Ferrer L Canine leishmaniosis - new concepts and insights on an expanding zoonosis: part one. Trends Parasitol (2008) 24(7):324–30. 10.1016/j.pt.2008.04.001 18514028

[2] Moreno J Alvar J Canine leishmaniasis: epidemiological risk and the experimental model. Trends Parasitol (2002) 18(9):399–405. 10.1016/s1471-4922(02)02347-4 12377257

[3] Baneth G Aroch I Canine leishmaniasis: a diagnostic and clinical challenge. Vet J (2008) 175:14–5. 10.1016/j.tvjl.2006.11.011 17215150

[4] Schaut RG Grinnage-Pulley TL Esch KJ Toepp AJ Duthie MS Howard RF Recovery of antigen-specific T cell responses from dogs infected with *Leishmania (L.) infantum* by use of vaccine associated TLR-agonist adjuvante. Vaccine (2016) 34(44):5225–34. 10.1016/j.vaccine.2016.09.016 PMC5053902 27665354

[5] Ashford DA David JR Freire M David R Sherlock I Eulálio MC Studies on control of visceral leishmaniasis: impact of dog control on canine and human visceral leishmaniasis in Jacobina, Bahia, Brazil. Am J Trop Med Hygiene (1998) 59(1):53–7. 10.4269/ajtmh.1998.59.53 9684628

[6] Sevá AP Ovallos FG Amaku M Carrillo E Moreno J Galati EA Canine-Based Strategies for Prevention and Control of Visceral Leishmaniasis in Brazil. PloS One (2016) 11(7):1–20. 10.1371/journal.pone.0160058 PMC4966914 27471852

[7] Pessoa-e-Silva R Vaitkevicius-Antão V Andrade T Silva ACO Oliveira GA Trajano-Silva LAM The diagnosis of canine visceral leishmaniasis in Brazil: Confronting old problems. Exp Parasitol (2019) 199:9–16. 10.1016/j.exppara.2019.02.012 30796913

[8] Reguera RM Morán M Pérez-Pertejo Y García-Estrada C Balaña-Fouce R Current status on prevention and treatment of canine leishmaniasis. Vet Parasitol (2016) 227:98–114. 10.1016/j.vetpar.2016.07.011 27523945

[9] Reis AB Giunchetti RC Carrillo E Martins-Filho OA Moreno J Immunity to Leishmania and the rational search for vaccines against canine leishmaniasis. Trends Parasitol (2010) 26(7):341–9. 10.1016/j.pt.2010.04.005. Cell Press. 20488751

[10] Costa CHN How effective is dog culling in controlling zoonotic visceral leishmaniasis? A critical evaluation of the science, politics and ethics behind this public health policy. Rev da Sociedade Bras Medicina Trop (2011) 44(2):232–42. 10.1590/s0037-86822011005000014 21468480

[11] Melo SN Teixeira-Neto RG Werneck GL Struchiner CJ Ribeiro RAN Sousa LR Prevalence of visceral leishmaniasis in A population of free-roaming dogs as determined by multiple sampling efforts: A longitudinal study analyzing the effectiveness of euthanasia. Prevent Vet Med (2018) 61:19–24. 10.1016/j.prevetmed.2018.10.010 30466654

[12] Romero GAS Boelaert M Control of Visceral Leishmaniasis in Latin America—A Systematic Review. PloS Neglect Trop Dis (2010) 4(1):584–600. 10.1371/journal.pntd.0000584 PMC2808217 20098726

[13] Werneck GL Costa CH de Carvalho FA Pires e Cruz Mdo S Maguire JH Castro MC Effectiveness of insecticide spraying and culling of dogs on the incidence of Leishmania infantum infection in humans: a cluster randomized trial in Teresina, Brazil. PloS Neglect Trop Dis (2014) 8:e3172. 10.1371/journal.pntd.0003172 PMC4214628 25357122

[14] Fernandes CB Magalhães Junior JT Jesus C Souza BMPS Laranjeira DF Fraga DBM Comparison of two commercial vaccines against visceral leishmaniasis in dogs from endemic areas: IgG, and subclasses, parasitism, and parasite transmission by xenodiagnosis. Vaccine (2014) 32:1287–95. 10.1016/j.vaccine.2013.12.046 24406392

[15] Moreno Assessment of Vaccine-Induced Immunity Against Canine Visceral Leishmaniasis. Front Vet Sci (2019) 6:168. 10.3389/fvets.2019.00168 31214607 PMC6558161

[16] Regina-Silva S Feres AMLT França-Silva JC Dias ES Michalsky EM de Andrade HM Field randomized trial to evaluate the efficacy of the Leish-Tec® vaccine against canine visceral leishmaniasis in an endemic area of Brazil. Vaccine (2016) 34(19):2233–39. 10.1016/j.vaccine.2016.03.019 26997002

[17] Bianciardi P Brovida C Valente M Aresu L Cavicchioli L Vischer C Administration of Miltefosine and Meglumine Antimoniate in Healthy Dogs: Clinicopathological Evaluation of the Impact on the Kidneys. Toxicol Pathol (2009) 37(6):770–5. 10.1177/0192623309344088 19690151

[18] Manna L Corso R Galiero G Cerrone A Muzj P Gravino AE Long-term follow-up of dogs with leishmaniosis treated with meglumine antimoniate plus allopurinol versus miltefosine plus allopurinol. Parasites Vectors (2015) 8(1):289. 10.1186/s13071-015-0896-0 26017164 PMC4458061

[19] Baneth G Shaw SE Chemotherapy of canine leishmaniosis. Vet Parasitol (2002) 106(4):315–24. 10.1016/s0304-4017(02)00115-2 12079737

[20] Proverbio D Spada E De Giorgi GB Perego R Failure of Miltefosine Treatment in Two Dogs with Natural Leishmania infantum Infection. Case Rep Vet Med (2014) 2014:1–7. 10.1155/2014/640151

[21] Foroughi-Parvar F Hatam G Vaccines for Canine Leishmaniasis. Adv Prevent Med (2014) 2014:1–15. 10.1155/2014/569193 PMC4297634 25628897

[22] Jain K Jain NK Vaccines for visceral leishmaniasis: A review. J Immunol Methods (2015) 422:1–12. 10.1016/j.jim.2015.03.017 25858230

[23] Magalhães FB Castro Neto AL Nascimento MB Santos WJT Medeiros ZM Lima Neto AS Evaluation of a new set of recombinant antigens for the serological diagnosis of human and canine visceral leishmaniasis. PloS One (2017) 12(9):e0184867. 10.1371/journal.pone.0184867 28957332 PMC5619722

[24] Moreno J Vouldoukis I Schreiber P Martin V McGahie D Gueguen S Primary vaccination with the LiESP/QA-21 vaccine (CaniLeish®) produces a cell-mediated immune response which is still present 1 year later. Vet Immunol Immunopathol (2014) 158:199–207. 10.1016/j.vetimm.2014.01.011 24560650

[25] Gradoni L Canine Leishmania vaccines: Still a long way to go. Vet Parasitol (2015) 208:94–100. 10.1016/j.vetpar.2015.01.003 25620293

[26] Alexander J Brombacher F T helper1/t helper2 cells and resistance/susceptibility to leishmania infection: is this paradigm still relevant? Front Immunol (2012) 3:80. 10.3389/fimmu.2012.00080 22566961 PMC3342373

[27] Bhowmick S Ravindran R Ali N IL-4 contributes to failure, and colludes with IL-10 to exacerbate *Leishmania donovani* infection following administration of a subcutaneous leishmanial antigen vaccine. BMC Microbiol (2014) 14(1):8–20. 10.1186/1471-2180-14-8 24428931 PMC3897895

[28] Oliveira GGS Magalhães FB Teixeira MCA Pereira AM Pinheiro CGM Santos LR Characterization of Novel *Leishmania infantum* Recombinant Proteins Encoded by Genes from Five Families with Distinct Capacities for Serodiagnosis of Canine and Human Visceral Leishmaniasis. Am J Trop Med Hygiene (2011) 85(6):1025–34. 10.4269/ajtmh.2011.11-0102 PMC3225146 22144438

[29] Vaughan S Kohl L Ngai I Wheeler RJ Gull K A repetitive protein essential for the flagellum attachment zone filament structure and function in *Trypanosoma brucei* . Protist (2008) 159:127–36. 10.1016/j.protis.2007.08.005 17945531

[30] Campos RM Mirna Nascimento M Ferraz C Pereira MMC Rocha PO Thompson GM Distinct mitochondrial HSP70 homologues conserved in various *Leishmania* species suggest novel biological functions. Mol Biochem Parasitol (2008) 160:157–62. 10.1016/j.molbiopara.2008.04.013 18541316

[31] Reis JC Estatística Aplicada à Pesquisa em Ciência Veterinária. Recife, Ed. Copyright (2003). 651 p.

[32] Massone F Anestesiologia Veterinária: Farmacologia e Técnicas: Texto e Atlas colorido. 5.ed. Rio de Janeiro: Guanabara Koogan (2008).

[33] Paiva-Cavalcanti M Brito MEF Souza WV Gomes YM Abath FGC The development of a real-time PCR assay for the quantification of *Leishmania infantum* in canine blood. Vet J (2009) 182(2):356–8. 10.1016/j.tvjl.2008.05.018 18603455

[34] Bradford MM A rapid and sensitive method for the Quantitation of Microgram Quantities of Protein Utilizing the Principle of Protein-Dye Binding. Anal Biochem (1976) 72:248–54. 10.1006/abio.1976.9999 942051

[35] Gonçalves-de-Albuquerque SC Oliveira CNC Vaitkevicius-Antão V Silva AC Luna CF Lorena VMB Study of association of the rs2275913 IL-17A single nucleotide polymorphism and susceptibility to cutaneous leishmaniasis caused by *Leishmania braziliensis* . Cytokine (2019) 123:154784. 10.1016/j.cyto.2019.154784 31344596

[36] Lorena VMB Verçosa AFA Machado RCA Moitinho-Silva L Cavalcanti MGA Silva ED Cellular immune response from chagasic patients to CRA or FRA recombinant antigens of *Trypanosoma cruzi* . J Clin Lab Anal (2008) 21:1–8. 10.1002/jcla.20209 PMC6649253 18348314

[37] Resende LA Roatt BM Aguiar-Soares RD Viana KF Mendonça LZ Lanna MF Cytokine and nitric oxide patterns in dogs immunized with LBSap vaccine, before and after experimental challenge with *Leishmania chagasi* plus saliva of *Lutzomyia longipalpis* . Vet Parasitol (2013) 198:371–81. 10.1016/j.vetpar.2013.09.011 PMC7115768 24129068

[38] Roatt BM Aguiar-Soares R Vitoriano-Souza J Coura-Vital W Braga SL Corrêa-Oliveira R Performance of LBSap Vaccine after Intradermal Challenge with L. infantum and Saliva of *Lu. longipalpis*: Immunogenicity and Parasitological Evaluation. PloS One (2012) 7(11):e49780. 10.1371/journal.pone.0049780 23189161 PMC3506642

[39] Saldarriaga OA Travi BL Park W Perez LE Melby PC Immunogenicity of a multicomponent DNA vaccine against visceral leishmaniasis in dogs. Vaccine (2006) 24:1928–40. 10.1016/j.vaccine.2005.10.052 16310897

[40] Costa-Pereira C Moreira ML Soares RP Marteleto BH Ribeiro VM França-Dias MH One-year timeline kinetics of cytokine-mediated cellular immunity in dogs vaccinated against visceral leishmaniasis. BMC Vet Res (2015) 11(92):1–10. 10.1186/s12917-015-0397-6 25880646 PMC4405846

[41] Martins VT Lage DP Duarte MC Costa LE Garde E Rodrigues MR A new Leishmania-specific hypothetical protein, LiHyT, used as a vaccine antigen against visceral leishmaniasis. Acta Tropica (2016) 154:73–81. 10.1016/j.actatropica.2015.11.006 26593442

[42] Santos FS Borja-Cabrera GP Miyashiro LM Grechi J Reis AB Moreira MAB Immunotherapy against experimental canine visceral leishmaniasis with the saponin enriched-Leishmune vaccine. Vaccine (2007) 25(33):6176–90. 10.1016/j.vaccine.2007.06.005 PMC7115527 17630055

[43] Matralis D Apadogiannakis E Kontos V Papadopoulos E Ktenas E Koutinas A Detection of intracellular IFN-c and IL-4 cytokines in CD4+ and CD8+ T cells in the peripheral blood of dogs naturally infected with *Leishmania infantum* . Parasite Immunol (2016) 38(8):510–15. 10.1111/pim.12335 27153749

[44] Rodríguez-Cortéz A Carrillo E Martorell S Todolí F Ojeda A Martínez-Flórez A Compartmentalized Immune Response in Leishmaniasis: Changing Patterns throughout the Disease. PloS One (2016) 11(5):e0155224. 10.1371/journal.pone.0155224 27171409 PMC4865036

[45] Srivastava S Shankar P Mishra J Singh S Possibilities and challenges for developing a successful vaccine for leishmaniasis. Parasites Vectors (2016) 9(277):1–15. 10.1186/s13071-016-1553-y 27175732 PMC4866332

[46] Maia C Campino L Cytokine and Phenotypic Cell Profiles of Leishmania infantum Infection in the Dog. J Trop Med 2012 (2012) 2012:541571. 10.1155/2012/541571 PMC3154519 21845197

[47] Solano-Gallego L Montserrrat-Sangrà S Ordeix L Martínez-Orellana P *Leishmania infantum*-specific production of IFN-γ and IL-10 in stimulated blood from dogs with clinical leishmaniosis. Parasites Vectors (2016) 9(317)1–10. 10.1186/s13071-016-1598-y PMC4893235 27260142

[48] Guedes RFM Bezerra BMO Lemos JC Nunes-Pinheiro DCS Hematologic and biochemical differentiation of dogs infected with *Leishmania infantum*, *Babesia* spp., *Dirofilaria* spp. and Cinomosis virus. Ciec Anim (2016) 26(3):37–51. 10.22456/1679-9216.82065

