# Supplementary figures and images for: Immunoprophylactic Potential of a New Recombinant Leishmania infantum Antigen for Canine Visceral Leishmaniasis: An In Vitro Finding
Source: Front Immunol. 2021 Jan 8;11:605044. doi: 10.3389/fimmu.2020.605044 (PMC7819978; doi:10.3389/fimmu.2020.605044)

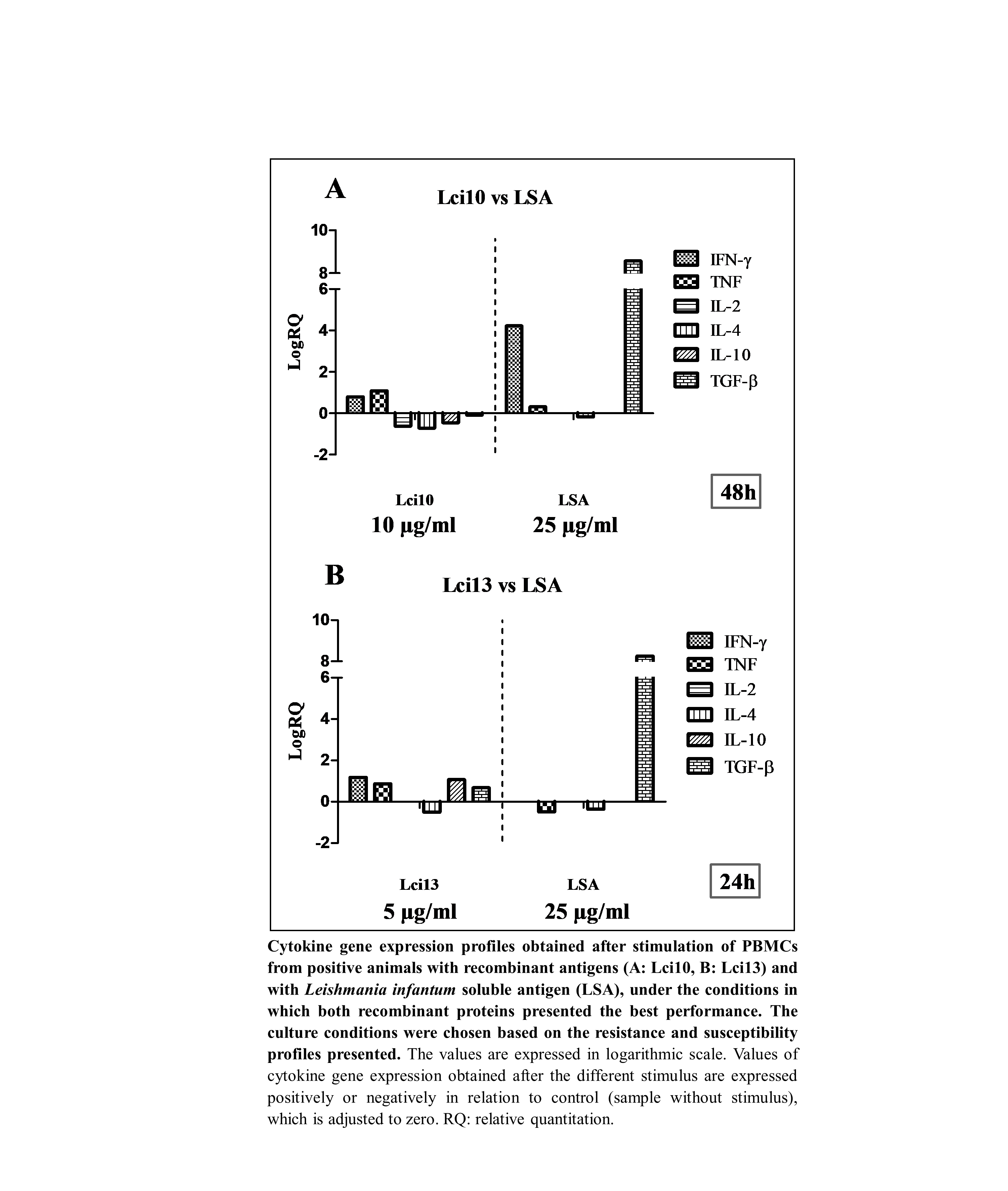

Supplement: Supplementary file 1 [file Image_1.tif]
